# Fusion of the *Mycobacterium tuberculosis* Antigen 85A to an Oligomerization Domain Enhances Its Immunogenicity in Both Mice and Non-Human Primates

**DOI:** 10.1371/journal.pone.0033555

**Published:** 2012-03-28

**Authors:** Alexandra J. Spencer, Fergal Hill, Jared D. Honeycutt, Matthew G. Cottingham, Migena Bregu, Christine S. Rollier, Julie Furze, Simon J. Draper, Karen C. Søgaard, Sarah C. Gilbert, David H. Wyllie, Adrian V. S. Hill

**Affiliations:** 1 The Jenner Institute, University of Oxford, Oxford, United Kingdom; 2 Imaxio SA, Lyons, France; University of Cape Town, South Africa

## Abstract

To prevent important infectious diseases such as tuberculosis, malaria and HIV, vaccines inducing greater T cell responses are required. In this study, we investigated whether fusion of the *M. tuberculosis* antigen 85A to recently described adjuvant IMX313, a hybrid avian C4bp oligomerization domain, could increase T cell responses in pre-clinical vaccine model species. In mice, the fused antigen 85A showed consistent increases in CD4^+^ and CD8^+^ T cell responses after DNA and MVA vaccination. In rhesus macaques, higher IFN-γ responses were observed in animals vaccinated with MVA-Ag85A IMX313 after both primary and secondary immunizations. In both animal models, fusion to IMX313 induced a quantitative enhancement in the response without altering its quality: multifunctional cytokines were uniformly increased and differentiation into effector and memory T cell subsets was augmented rather than skewed. An extensive *in vivo* characterization suggests that IMX313 improves the initiation of immune responses as an increase in antigen 85A specific cells was observed as early as day 3 after vaccination. This report demonstrates that antigen multimerization using IMX313 is a simple and effective cross-species method to improve vaccine immunogenicity with potentially broad applicability.

## Introduction

A major challenge in vaccinology is the development of effective vaccines against intracellular pathogens where cell mediated immunity plays an important protective role. Viral vectored vaccines have a remarkable capacity to induce and boost antigen-specific T cells [1], but higher frequency responses will likely be required to achieve useful protective efficacy [2]. There is, therefore, a need for adjuvants in the next generation of vectored vaccines to increase T cell immunogenicity.

Oligomerization is employed by many natural proteins to increase protein valency, binding affinity and structural stability [3], and while a pentameric coiled coil was initially used to improve B-cell responses in mice [4] its sequence is too similar to its human ortholog to be considered safe for use in humans. Recently, a series of homologous oligomerization protein domains were shown to act as adjuvants in mice, resulting in an augmentation of both B and T cell responses [5], [6]. These proteins were derived from the domain encoded by the last exon of the complement 4 binding protein (C4bp) α-chain. This exon encodes the only domain not involved in the complement related functions of C4bp and is both essential and sufficient for oligomerization of the seven C4bp alpha chains [7]# and a number of other proteins [8], [9], [10], [11], [12]. Fusion of recombinant *Plasmodium yoelii* MSP-1_19_ protein to C4bp oligomerization domains from a variety of mammalian and avian species was shown to improve antibody responses to this weak immunogen [6]; of the domains tested, a chicken C4bp hybrid with less than 20% homology to human C4bp, called IMX313, was shown to induce the highest titers of MSP-1_19_ specific antibodies, buts its effect on T cell responses was not investigated.

Tuberculosis (TB) remains one of the most serious worldwide infections despite the use of the *M. bovis* strain Bacillus Calmette-Guérin (BCG) as a vaccine since the 1920s. The most advanced sub-unit vaccine in clinical development is a modified vaccinia virus Ankara (MVA) expressing the *M. tuberculosis* protein 85A. Clinical trials in both the UK and Africa have shown the substantial capacity of MVA-Ag85A to boost T cell responses to BCG in healthy individuals [13], [14], [15], whether this capacity is maintained in HIV-infected individuals is unclear. Although there is currently no clear correlate of protection, T cells have been shown to play an important role [16], [17], [18] and therefore strategies to increase the level of vaccine induced T cells could have a substantial impact.

In this study we have assessed whether fusion to the IMX313 domain could enhance the T cell mediated response to the *M. tuberculosis* antigen 85A in two pre-clinical animal species, mice and rhesus macaques. In both animal models, IFN-γ ELISpot and multi-parameter flow cytometry were used to investigate the effects of fusion to IMX313 on the overall quality of the immune response in terms of cytokine secretion and generation of effector or memory T cell subsets. Fusion of antigens to the IMX313 domain is a straightforward method and its ability to enhance T cell immune responses could have broad applicability across a variety of animal species and disease settings. In the first instance, we intend to undertake a direct comparison in humans of MVA-Ag85A with MVA-Ag85A IMX313 to confirm the results described here in two very distinct animal species.

## Results

### Fusion to IMX313 multimerizes antigen 85A

**Figure 1:**
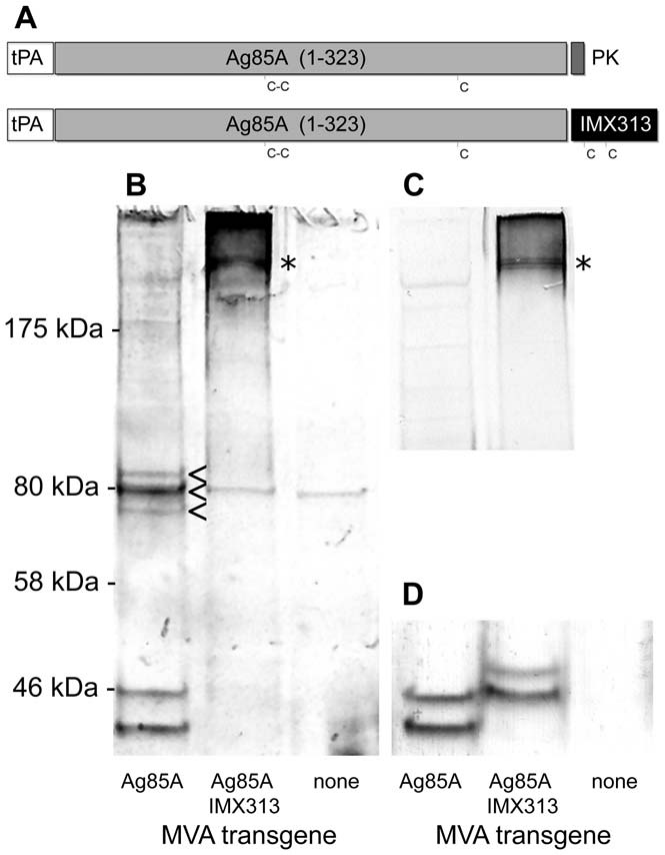
Schematic representation of Ag85A and Ag85A IMX313 protein constructs. (**A**). tPA = signal peptide from human tissue plasminogen activator (30 aa); Ag85A(1–323) = residues 1 to 323 of *M. tuberculosis* antigen 85A; PK = SV5-PK epitope tag (9 aa); IMX313 = hybrid chicken oligomerization domain of C4bp (55 aa). C indicates cysteine residue. Western blots labeled with anti-Ag85A polyclonal antibody (**B** and **D**) or anti-IMX313 polyclonal antibody (**C**) of non-reducing (**B** and **C**) and reducing (**D**) SDS-PAGE gels of BHK cell lysates 24 h post infection with the indicated viruses. Arrowheads indicate disulphide-linked dimers formed by Ag85A and asterisks indicate higher molecular weight disulphide-linked multimers, presumably heptamers, formed by Ag85A IMX313.

To confirm that fusion of IMX313 resulted in formation of disulphide-linked multimers without affecting protein expression, cell lysates from MVA infected BHK cells were analyzed by Western blots of reducing and non-reducing SDS-PAGE gels (Figure 1). Under reducing conditions, both Ag85A and Ag85A IMX313 proteins migrated at the expected apparent molecular weight of the mature monomeric peptide (35.5 kDa for Ag85A or 40.4 kDa for Ag85A IMX313) (Figure 1d). For both proteins, an additional higher molecular weight band (∼45 kDa for Ag85A and ∼50 kDa for Ag85A IMX313) was observed, probably representing a glycosylated species, since a predicted N-linked glycosylation site (NNT) is present within Ag85A (http://www.cbs.dtu.dk/services/NetNGlyc/), though this conclusion has not been experimentally verified. More importantly, when the reducing agent was omitted (Figure 1b and 1c), the Ag85A IMX313 fusion protein migrated exclusively in excess of ∼200 kDa, consistent with formation of the predicted heptamers (248 kDa). Although other higher-order species were observed, these results indicate that the IMX313 domain was functioning as expected to induce antigen multimerization.

### Fusion of antigen 85A to IMX313 enhances CD4^+^ and CD8^+^ T cell responses in mice

**Figure 2:**
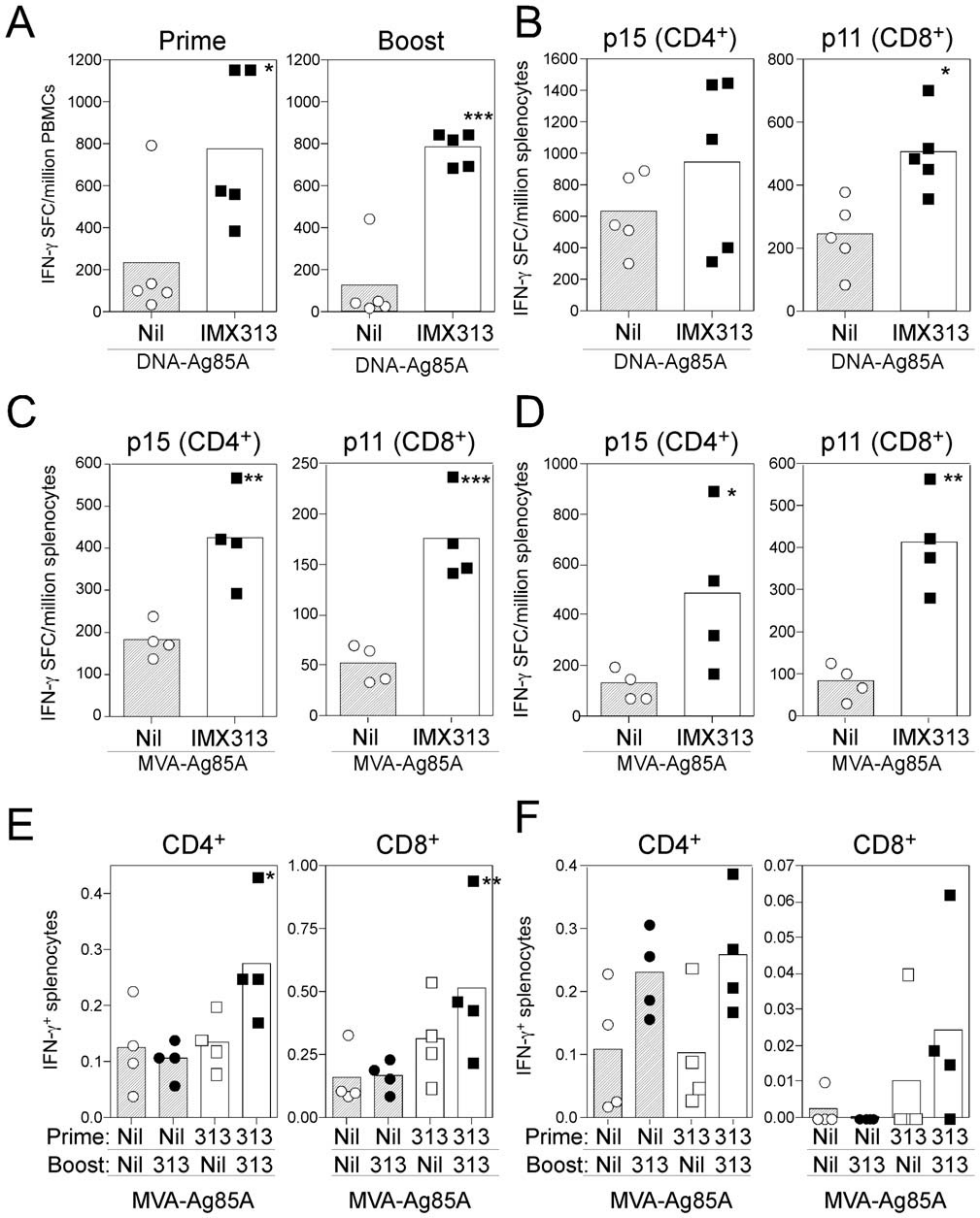
Screening of DNA and MVA vaccines in mice. **A** & **B**) Balb/c mice were immunized i.m. at weeks 0 and 2 with 50 µg of pSG2-Ag85A or pSG2-Ag85A IMX313. IFN-γ ELISpot was used to measure the response to the p15 and p11 peptides (together) in the blood 12 days after each vaccination (**A**) of each individual peptide in the spleen 14 days after the final vaccination (**B**). **C** & **D**) In two separate experiments, Balb/c mice were immunized i.m. (**C**) or i.d. (**D**) with 10^6^ PFU of MVA-Ag85A or MVA-Ag85A IMX313 and the response to p15 and p11 measured in the spleen of all animals 7 days later. **E** & **F**) Balb/c (**E**) or C57BL/6 (**F**) mice were immunized i.m. with 10^6^ PFU of MVA-Ag85A or of MVA-Ag85A IMX313 at weeks 0 and 2. Intracellular cytokine staining was performed on spleen samples taken 2 weeks after the final vaccination. Graphs represent the frequency of IFN-γ producing CD4^+^ (left) or CD8^+^ (right) T cells in response to a single pool containing all Ag85A peptides. For all graphs, the bar represents the mean per group with each individual animal displayed as a single point. Data were analyzed with a 2-way analysis of variance; fusion to IMX313 was shown to induce a significant effect in all data sets (**A** p<0.001, **B** p = 0.0298, **C** p<0.001, **D** p = 0.0002, **E** p = 0.0017, **F** p = 0.0415). No interaction between prime/boost or CD4^+^/CD8^+^ T cell responses were observed in any experiment and when analyzed with a post-hoc Bonferroni test; the asterisks denote the level of statistical significance (*p<0.05, **p<0.01, ***p<0.001).

Initial screening experiments were performed in mice to assess the ability of IMX313 to enhance antigen 85A-specific responses. Balb/c mice received two intramuscular (i.m.) immunizations with either 50 µg of DNA-Ag85A or DNA-Ag85A-IMX313 two weeks apart and the immune response to Ag85A measured in the blood or spleen by IFN-γ ELISpot. Following a single immunization of 50 µg DNA plasmid vaccine, significant increases in the response to the dominant CD4^+^ (p15) and CD8^+^ (p11) T cell epitopes were observed in PBMCs from mice vaccinated with the Ag85A IMX313 compared to Ag85A vaccinated controls (Figure 2A). These increases were enhanced after a second homologous DNA immunization (Figure 2A) and a significant increase in the p11-specific response was observed in *ex vivo* splenocytes two weeks after the final vaccination (Figure 2B) (2.1-fold increase). A small 1.5-fold increase in the *ex vivo* p15 response was also detected but did not reach statistical significance (Figure 2B).

For the assessment of IMX313 in an MVA vector, Balb/c mice were vaccinated intramuscularly (i.m.) or intradermally (i.d.) with 10^6^ PFU of either MVA-Ag85A or MVA-Ag85A IMX313 and the immune responses measured in the spleen of mice by IFN-γ ELISpot (Figure 2C and D). One week after i.m. vaccination, significant increases in both p15 (2.3-fold) and p11 (3.3-fold) specific responses were observed in MVA-Ag85A IMX313 vaccinated mice compared to MVA-Ag85A controls (Figure 2C), with a similar enhancement observed in intradermally (i.d.) immunized mice (Figure 2D). MVA vaccines were subsequently compared in a variety of prime-boost combinations to determine the priming versus boosting capacity of the IMX313 fusion in two separate strains of mice: Balb/c and C57BL/6. Mice received an i.m. priming vaccination with either MVA-Ag85A or MVA-Ag85A IMX313 followed two weeks later with either 10^6^ PFU of MVA-Ag85A or MVA-Ag85A IMX313 delivered via the i.m. route (Figure 2E and F). Two weeks after the second immunization, the highest response in either strain of mice was observed after homologous immunization with MVA-Ag85A IMX313 (Figure 2E and 2F). In Balb/c mice, a 2.2-fold increase in the mean CD4^+^ T cell response (95% CI 1.82–2.60) and 3.3-fold increase in the mean CD8^+^ T cell response (95% CI 2.52–4.00) was observed when compared to mice homologously vaccinated with MVA-Ag85A (Figure 2E). In summary, fusion of IMX313 to antigen 85A consistently enhanced T cell responses in the two vaccine delivery systems and in two strains of mice.

### Fusion of antigen 85A to IMX313 enhances the immune response in rhesus macaques

While mice have proved to be excellent indicators of immunogenicity, the ability to predict efficacy from murine tuberculosis models has often been disappointing [19] and larger animals may be better predictors of human immunogenicity and efficacy. As the primary focus of this study was to test the capacity of IMX313 to increase T cell responses, we performed further immunological assessments in non-human primates.

**Figure 3:**
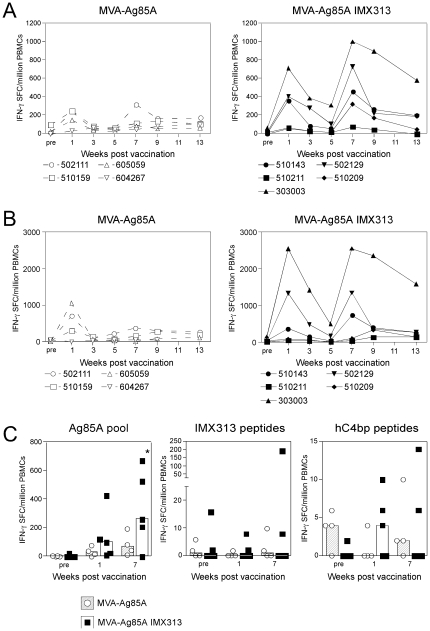
Macaque IFN-γ ELISpot responses. Male rhesus macaques were immunized i.d. at weeks 0 and 6 with either 10^8^ PFU MVA-Ag85A or MVA-Ag85A IMX313 and the response to antigen 85A measured in the blood by IFN-γ ELISpot before vaccination (pre) and every 2 weeks from week 1 onwards. Graphs represent the response of each individual animal to a single pool containing all antigen 85A peptides (**A**) or the sum of 7 separate peptide pools (**B**). **C**) Graphs represent the ELISpot responses of frozen PBMCs to a pool of antigen 85A peptides, a pool containing IMX313 peptides and a pool containing hC4bp peptides, pre-vaccination and at week 1 or week 7 post-vaccination. Bars represent the median group response with each animal displayed as a single point. Data was analyzed with a 2-way analysis of variance with a post-hoc Bonferroni test; the asterisks denote the level of statistical significance (*p<0.05).

Rhesus macaques were immunized with 10^8^ PFU of MVA-Ag85A or MVA-Ag85A IMX313 at weeks 0 and 6, and the response to antigen 85A was measured by IFN-γ ELISpot 1 week after vaccination, then every 2 weeks using a single pool of 66 peptides (Figure 3A) and 7 pools of 20 peptides each (Figure 3B). The peak of the antigen 85A-specific responses was observed 1 week after each vaccination (Figure 3A and B) consistent with previous data in mice and humans [20]. At multiple timepoints, higher antigen 85A responses were observed in the group of animals vaccinated with the MVA-Ag85A IMX313 when compared to those vaccinated with MVA-Ag85A (Figure 3). We conducted two separate analyses to determine if there was a statistically significant effect of IMX313. Firstly, without making distributional assumptions, we noted that over the weeks 1–13, the total number of Ag85A IFN-γ secreting cells in the MVA-Ag85A IMX313 group was greater than those in the MVA-Ag85A control group (Kruskal-Wallis test, p = 0.02), this was also true with single peptide pools A, B, C, D, and G (p = 0.05, 0.07, 0.05, 0.08, 0.02, respectively, Figure S1). In a second approach, we constructed a parametric model of the responses to the seven individual peptide pools to derive an estimate of IMX313 induced effect of 1.8-fold (95% CI 1.31–2.41). Despite the increased variance in immune responses to Ag85A detected in non-human primates, the magnitude of the increase was similar in the two species.

To measure whether vaccination with IMX313 induced cross-reactive T cells to C4bp, frozen PBMCs were thawed and the response to 85A peptides, IMX313 peptides and hC4bp peptides measured by IFN-γ ELISpot. In agreement with the data from freshly isolated PBMCs, a statistically significant increase in the antigen 85A response was observed 1 week after the second vaccination in MVA-Ag85A IMX313 animals compared to the controls (Figure 3C left panel). Only 1 animal had a detectable T cell response to IMX313 peptides (Figure 3C central panel) and importantly no detectable response to human C4bp was observed in any of the macaques (Figure 3C right panel). Antibodies to 85A, IMX313 and human C4bp were also assessed but no detectable responses above background to any of the antigens were observed (data not shown).

In summary, consistent with the data observed in mice, fusion to IMX313 resulted in an overall increase in the responses to the antigen 85A induced by MVA vaccination and importantly did not result in the induction of cross-reactive T cells to the oligomerization domain of C4bp.

### Fusion of antigen 85A to IMX313 does not alter the quality of the immune response in mice and macaques

A number of recent studies have suggested that the quality of the immune response, as determined by cytokine polyfunctionality, can have a substantial effect on the overall protective ability of a vaccine regime [21], [22]. While there is no clear correlate of protection to TB, T cells have a protective effect [16], [17], [18] and any second-generation TB vaccine should ideally augment the level of both CD4^+^ and CD8^+^ T cell immunogenicity while maintaining its overall quality.

**Figure 4:**
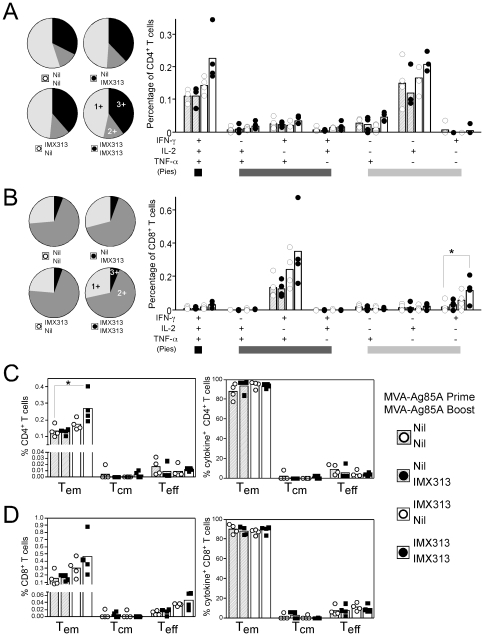
Quality of the antigen 85A response in Balb/c mice. **A** & **B**) Pie charts represent the proportion of CD4^+^ T cells (**A**) or CD8^+^ T cells (**B**) secreting either 1, 2, or all 3 cytokines measured, while the graphs represent the percentage of each cytokine subset as a frequency of all CD4^+^ or CD8^+^ T cells. **C** & **D**) Any CD4^+^ (**C**) or CD8^+^ (**D**) T cell secreting either IFN-γ, TNF-α or IL-2 was further subdivided into either CD62L^−^,CD127^−^ T_eff_, CD62L^−^,CD127^+^ T_em_ or CD62L^+^, CD127^+^ T_cm_ cells and plotted as a percentage of the total CD4^+^ or CD8^+^ T cell population (left panel) or as a percentage of all cytokine positive cells (right panels). Bars represent the group mean response with each individual animal displayed as a single point. Data were analyzed with a 2-way analysis of variance; fusion to IMX313 was shown to induce a significant effect only for the absolute frequency of cytokine^+^ CD8^+^ T cells (**B** graph p = 0.0145) and absolute frequency of memory subset CD4^+^ T cells (**C** left panel p = 0.0337). No interaction between prime-boost combination or cytokine subset was observed in any experiment and when analyzed with a post-hoc Bonferroni test; the asterisks denote the level of statistical significance (*p<0.05).

We therefore investigated whether fusion to IMX313 altered the quality of the immune response in terms of cytokine secretion and differentiation into effector and memory T cells. In the MVA prime-boost experiments described above (Figure 2E), splenocytes were stimulated with a single pool of antigen 85A analyzed for the intracellular production of IFN-γ, IL-2, TNFα and simultaneously stained for CD62L and CD127 to discriminate between the three main population of T effector and memory cells [23], [24]. In Balb/c mice, CD4^+^ T cells were shown to produce predominately all of the three cytokines measured (IL-2, IFN-γ and TNF-α: triple positive cells) or IL-2 alone (Figure 4A) and homologous prime-boost with MVA-Ag85A IMX313 was shown to induce the highest frequency of both of these subsets when compared to the control MVA-Ag85A vaccinated group (Figure 4A). This increase in frequency did not come at a cost to other cytokine producing subsets, as there was no change in the proportion of cytokine producing cells that secreted either 1, 2 or all 3 cytokines (Figure 4A pie-charts). CD8^+^ T cells demonstrated a difference in the cytokine profile compared to CD4^+^ T cells, the predominant cytokine producing population observed was CD8^+^ T cells that simultaneously produced IFN-γ and TNF-α (double positive) or IFN-γ only (Figure 4B). An overall increase in the frequency of IFN-γ and TNF-α or IFN-γ only producing CD8^+^ T cells in MVA-Ag85A IMX313 vaccinated mice did not alter the other subsets, as equal proportions of cells producing 1, 2 or all 3 cytokines were observed between all groups of mice (Figure 4B pie-charts). To analyze the differentiation of antigen-specific cells into effector and memory T cell subsets, any cell producing at least one cytokine (*i.e.* IFN-γ or TNF-α or IL-2) was analyzed for the expression of surface markers of CD127 and CD62L and separated into CD127^+^, CD62L^+^ T central memory (T_cm_) cells, CD127^+^, CD62L^−^ T effector memory (T_em_) cells or CD127^−^, CD62L^−^ T effector (T_eff_) cells. A higher frequency of both CD4^+^ T_em_ cells (Figure 4C) and CD8^+^ T_em_ cells (Figure 4D) were observed in MVA-Ag85A IMX313 vaccinated mice when compared to MVA-Ag85A controls (Figure 4C). In all groups of animals, around 90% of either CD4^+^ or CD8^+^ cytokine producing T cells displayed a T_em_ phenotype, indicating that the fusion of antigen 85A to IMX313 did not skew the distribution of measured phenotypes.

**Figure 5:**
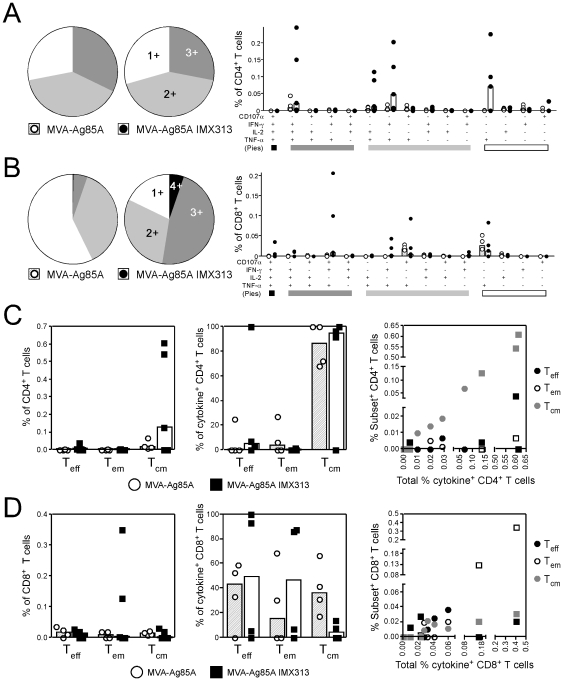
Quality of the response to antigen 85A in rhesus macaques 1 week after a homologous MVA-Ag85A boost. **A** & **B**) Graphs represent the frequency of each cytokine producing subset as a percentage of all CD4^+^ (**A**) or CD8^+^ (**B**) T cells, while the pie charts represent the proportion of cytokine producing cells positive for 1, 2, 3 or all 4 markers. **C** & **D**) Any cell positive for either CD107a, IFN-γ, TNF-α or IL-2 was further subdivided into a CD28^+^CD95^−^ T_eff_, CD28^−^CD95^−^ T_em_ or CD28^+^CD95^+^ T_cm_ cell. Graphs on the left represent the absolute frequency of each memory population relative to the total number of CD4^+^ (**C**) or CD8^+^ (**D**) T cells, centre graphs represent the percentage of each population relative to cytokine producing cells, and graphs on the right represent total percentage of cytokine^+^ cells compared to the percentage of each individual T cell population. For each graph the bars represent the median group response, with each animal displayed as a single point. Data were analyzed with a 2-way analysis of variance, no statistical difference between the two groups of vaccinated animals was observed.

A similar analysis of quantity versus quality was performed on the non-human primate samples one week after the second MVA vaccination. Frozen PBMCs were restimulated with a single pool of antigen 85A peptides and cells analyzed for upregulation of CD107 (a marker of degranulation) [25], intracellular production of IFN-γ, IL-2 and TNF-α and surface expression of CD95 and CD28. The predominant CD4^+^ T cell sub-population seen was cells secreting TNF-α alone or in combination with IFN-γ, IL-2 or both (*i.e.* triple positive cells) (Figure 5A). Consistent with the data in mice, MVA-Ag85A IMX313 vaccinated animals displayed an overall increase in the frequency of these populations, but this did not result in a change in the proportion of cells producing either 1, 2 or all 3 cytokines (Figure 5A pie-charts). Substantially lower overall responses were observed in CD8^+^ T cells (Figure 5B graphs) and in the MVA-Ag85A IMX313 group, two strong responders drove a shift towards a higher proportion of cells producing all three cytokines and upregulating CD107a (Figure 5B graphs). Importantly, the weaker responding animals in this group displayed an identical proportional distribution to animals vaccinated with MVA-Ag85A (Figure 5B pie-charts), suggesting it is the overall size of the immune response and not an effect of IMX313 that was driving an increase in CD8^+^ T cell multi-functionality. At the same time, any cell producing one or more cytokines or CD107a was analyzed for expression of CD28 and CD95 and further subdivided into CD28^+^CD95^+^ T_cm_, CD28^−^CD95^+^ T_em_ or CD28^+^CD95^−^ T_eff_ cells [26], [27], [28]. One week after a second vaccination with MVA, T_cm_ was the predominant phenotype of antigen 85A specific CD4^+^ T cells observed (Figure 4C), consistent with previous data from macaques [28]. An increase in the total frequency of CD4^+^ T_cm_ cells was seen in MVA-Ag85A IMX313 vaccinated animals (Figure 5C left panel) yet this did not alter the differentiation of cells into either T_eff_, T_em_ or T_cm_ (Figure 5C middle panel). This trend was less clearly observed for CD8^+^ T cells due to the overall lower responses (Figure 5D). However it is clear that strong CD8^+^ T cell responding animals displayed a predominantly T_em_ phenotype (Figure 5D right panel), while weaker responders displayed equal distribution of cells into T_eff_, T_em_ and T_cm_ cells, regardless of the vaccine regime (Figure 5D). In summary, the data from both mice and macaques demonstrate that fusion to IMX313 increased the frequency of antigen 85A specific cells without altering the overall quality of the immune response generated in terms of multi-functionality or differentiation into effector or memory T cell subsets.

### IMX313 has a very early effect on the immune response

An increase in antigen persistence, stability and/or uptake as a result of multimerization or binding of IMX313 to a specific (*in vivo*) target receptor, are all conceivable mechanism of IMX313 induced immune response enhancement. As all of these mechanisms could have an early effect on the immune response, we characterized the *in vivo* response as early as 3 days after MVA vaccination.

We then characterized the cellular composition of the lymph node to determine whether IMX313 was mediating an effect on APC recruitment and activation. Three days after MVA vaccination, draining auricular lymph nodes were harvested to investigate the frequency and number of CD4^+^ T cells, CD8^+^ T cells, B cells, macrophages and more specifically a number of different dendritic cell (DC) subsets (Figure S2A). At this timepoint, there were significant changes in the composition of draining lymph nodes in terms of the frequency of each cell population (Figure S2B), but all lymphocyte populations where shown to increase in total number (Figure S2C). The most dramatic increase was observed for CD11c^+^ DCs (Figure S2C) primarily due to an influx in CD11b^+^ MHC II^intermediate^ (^int^) DCs (Figure S2D), although other DC subsets did also increase in number (Figure S2D, E). Recruitment of these DCs was antigen 85A independent, as a similar frequency and number were present after vaccination with an irrelevant MVA (Figure S2F, G). IMX313 did not however alter the activation state of CD11b^+^ MHC II^int^ DCs, as no change in CD80 (Figure S2H) or MHC class II expression (Figure S2I) was observed.

**Figure 6:**
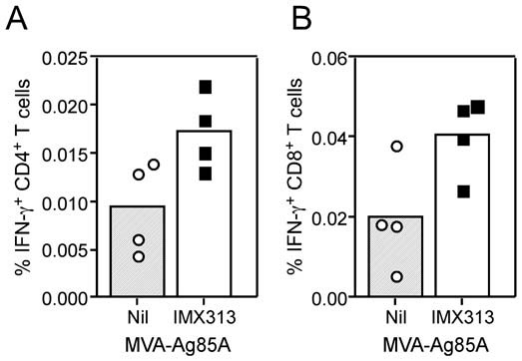
T cell responses in the draining auricular draining lymph node three days after vaccination with 10^6^ PFU MVA-Ag85A. Graphs represent the frequency of IFN-γ^+^ CD4^+^ T cells (**A**) or CD8^+^ T cells (**B**) after 6 hours of restimulation with the total antigen 85A peptide pool and staining for a variety of surface activation markers (S4). Data were log transformed and analyzed with a 2-way analysis of variance; a significant effect of IMX313 fusion was only observed for the frequency of antigen 85A specific cells p = 0.0101 but not for any surface marker (Figure S3).

While we observed no prolonged antigen 85A persistence or specific effect of IMX313 on lymph node cell populations, these experiments did not rule out an early effect in the initial *in vivo* antigen dose, which could also be affected by stability and uptake of antigen 85A. As changes in T cell frequencies and activation marker expression has been observed *in vivo* with alterations in antigen dose [29], [30], we investigated both of these factors as a potential surrogate marker of *in vivo* antigen dose. Balb/c mice were immunized with MVA-Ag85A or MVA-85A IMX313 and draining lymph nodes harvested 3 days later. Using intracellular IFN-γ staining to identify antigen 85A-specific cells, we observed an early increase (2-fold) in the frequency of both CD4^+^ (Figure 6A) and CD8^+^ (Figure 6B) T cells in MVA-Ag85A IMX313 vaccinated mice compared to MVA-Ag85A controls. Despite this increase, we observed no statistically significant changes in the levels of a number of T cell activation markers investigated (Figure S3), demonstrating once again that IMX313 was not skewing the induction of the T cell responses in any particular way.

In summary, while this *in vivo* characterization did not suggest a specific receptor for IMX313, we observed an increase in antigen specific cells as early as three days after MVA vaccination, showing that the effect of IMX313 occurs very early in the immune response.

## Discussion

In this study we have used the *M. tuberculosis* antigen 85A as a model to demonstrate the potent cross-species adjuvant effect of IMX313. Straightforward procedures were used to incorporate this adjuvant into two vectored vaccine systems, and an immune-potentiating effect was subsequently demonstrated in both mice and non-human primates. In both animal models the fusion of antigen 85A to IMX313 resulted in an augmentation of the response without skewing it towards a particular cytokine-producing population as has been observed with some traditional adjuvants [31], [32].

Our detailed *in vivo* characterization of the response three days after vaccination did not reveal a specific activation effect of IMX313. However at this timepoint, we observed a significant increase in the frequency of antigen 85A specific CD4^+^ and CD8^+^ T cells (Figure 6). The two-fold increase at day 3 was equivalent to the fold increase observed at day 7, and since this fold increase was maintained over the course of the response, it would suggest that IMX313 does not affect the rate of T cell division. Therefore the increase in antigen specific cells occurs as a result of an early event in T cell activation, most likely an increase in the effective *in vivo* antigen dose, *i.e.* amount of antigen presented. This is supported by our data showing no shift in the kinetics or quality of the 85A immune response by fusion to IMX313. In addition, the consistent effect of IMX313 in DNA and MVA and *via* different vaccination routes would support a mechanism that relies on an early antigenic effect, as opposed to a specific recruitment of cells or receptors that may not be accessed by different vectors or vaccination routes.

It is not clear how multimerization enhances the effective *in vivo* antigen dose. Fusion to C4bp was originally proposed to increase the serum half-life of proteins and their immunogenicity [6], but multimerization may itself result in an increase the uptake of antigen 85A by APCs due to increased avidity. Antigen multimerization may also lead to prolonged antigen presentation by making antigen 85A more difficult to process [33]. In an elegant study, Delmarre et al demonstrated that by increasing the lysosomal susceptibility, both T and B cell immunogenicity was reduced *in vivo*. Bins et al [34]# subsequently demonstrated that increasing *in vivo* luciferase stability resulted in increased levels of T cell immunogenicity induced by DNA vaccination without altering the kinetics of the immune response, supporting the data observed in this study. Fusion of antigen 85A to IMX313 may either increase serum half-life, improve antigen uptake, prolong antigen processing or all of the above and further experiments are underway to try and address some of these possibilities.

While many molecular adjuvants fall victim to species differences in signaling and activation pathways, multimerization takes advantage of the immune system's natural response to an increase in effective *in vivo* antigen dose, so that its effect is consistently observed through different vaccine platforms and in two very different animals species. Thus multimerization as an adjuvant platform should have broad applicability to a variety of diseases where cell-mediated immunity plays an important role, *e.g.* malaria, TB, HIV and influenza. In summary, this relatively simple technology of antigen multimerization could be useful in a wide range of vaccine programs, both veterinary and human, as well as for some non-communicable diseases.

## Materials and Methods

### Plasmids, viruses and cells

A 229 bp DNA fragment encoding the IMX313 domain was PCR amplified from the plasmid pIMX313 [6] using the oligonucleotides 5′-GAAGCCCGACCTGCAACGTGGATCCAAGAAGCAAGGTGATGCTGATG-3′ and 5′-AGGGCCCTCTAGATGCATGCTCGAGCGGCCGCTTATTACTCCTTGCTCAGTCCTTGC-3′ and inserted into the DNA vaccine vector pSG2-Ag85A [35] using site-directed mutagenesis [36]#. This replaced the 9 amino acid PK tag at the C-terminus of antigen 85A protein with the 55 amino acid IMX313 domain. Ag85A IMX313 was conventionally subcloned into the poxvirus shuttle vector pMVA-GFP-TD which places it under the control of the vaccinia virus p7.5 promoter and provides flanks for recombination at the TK locus of MVA. Markerless recombinant viruses were isolated following transfection of this plasmid into infected primary chick embryo fibroblasts (CEFs) (Day 9 of gestation) (Institute for Animal Health) by transient dominant selection with GFP. For immunizations, viruses were amplified in CEFs, purified over sucrose cushions, and titered twice in duplicate by plaque assay according to standard methods. Virus identity and purity was confirmed by PCR.

### Ethics Statement

Mice were used in accordance with the UK Animals (Scientific Procedures) Act under project license number 30/2414 granted by the UK Home Office.

Male rhesus macaques (*Macaca mulatta*) aged between 2 ½ to 6 years were maintained and used at the Institute of Neurobiology and Molecular Medicine, National Research Council of Italy, in accordance with approval granted by the ethics committee of the Italian Ministry of Health (Decree by low n°: 2007 114 B and n° 2007 115 C). The animal care routine and experimental procedures were in compliance with national and international laws and policies (EEC Council Directive 86/609; Italian Legislative Decree 116/92). Animal welfare was ensured and steps taken to ameliorate suffering in accordance with the recommendations of the Weatherall report, “The use of non-human primates in research.” To minimize distress and suffering related to vaccination or blood withdrawal, animals were anesthetized by i.m. injection of 10 mg/kg ketamine hydrochloride for all procedures and no animals were sacrificed at the end of the experiment.

### Animals and Immunizations

Female Balb/c or C57BL/6 mice of 6 weeks of age or older (Harlan, UK) were immunized i.m. in the musculus tibialis or intradermally (i.d.) in the ear with a total volume of 50 µl of vaccine diluted in PBS. Mice received either 50 µg of DNA or 10^6^ PFU of MVA per vaccination.

Rhesus Macaques were assigned into each group to ensure homogeneous distribution of age and body weight. The control group (MVA-Ag85A) included a total of 4 animals with an average weight of 5 kg (range 3.9–7.5 kg) and age of 4 years (range 2.8–5.9); the test group (MVA-Ag85A IMX313) included 5 animals with an average weight of 4.98 kg (range 4–7.55) and average age of 4 years (range 3.4–6). Animals received two intradermal immunizations, at weeks 0 and 6, with of 10^8^ PFU of MVA, with arms switched between vaccinations. Blood samples were kept at room temperature and shipped overnight to Oxford where all processing and assays were performed.

### Western Blots

Monolayers of BHK-21 cells (European Collection of Cell Cultures) were infected for 24 hours with MVA-Ag85A, MVA-Ag85A IMX313 or an unrelated recombinant MVA at 4 PFU per cell. Cells were washed in PBS and harvested into non-reducing Laemmli's sample buffer. After addition of ß-mercaptoethanol to half of each sample, the lysates were processed for SDS-PAGE and Western blotting by standard methods. Lanes were loaded with volumes of lysate corresponding to approximately 10^5^ infected cells. Blots were labeled with chicken anti-85A (AbCam) or a hyperimmune serum from rabbits immunized with IMX313 protein, followed by alkaline phosphatase conjugated secondary antibodies (Jackson) and colorimetric visualization.

### Peptides

Murine cells were stimulated with the CD8 epitope p11 WYDQSGLSV (aa 103–111), CD4 epitope p15 TFLTSELPGWLQANRHVKPT (aa 142–161) (ProImmune, UK) from Ag85A (GenBank accession no. CAA17868) [37], [38], [39], [40] or a single pool containing all 66 antigen 85A peptides (20mer peptides overlapping by 10) (Peptide Protein Research, UK). Fresh and frozen macaque PBMCs were stimulated with the single pool of antigen 85A peptides, 7 pools spanning antigen 85A (Table S1), a single pool of IMX313 peptides (Table S2) (20mers overlapping by 10) (ProImmune) or a single pool of the oligomerization domain of human C4bp (Table S2) (20mers overlapping by 10) (ProImmune) at a final concentration of 2 µg/ml.

### ELISpots

Murine spleens or peripheral blood samples were treated with ACK lysis buffer to remove RBCs prior to stimulation with the relevant peptides (final concentration of 2 µg/ml) on IPVH-membrane plates (Millipore) coated with 5 µg/ml anti-mouse IFN-γ (AN18). Macaque PBMCs were isolated over a lymphoprep gradient (Sigma) while frozen PBMCs were thawed and rested overnight in media containing 10 U/ml Benzonase (Sigma). 250 000 PBMCs per well were stimulated with relevant peptides at a final concentration of 2 µg/ml on IPVH-membranes coated with 15 µg/ml anti-human IFN-γ (GZ4).

After 18–20 hours of stimulation, IFN-γ spot forming cells (SFC) were detected by staining membranes with either anti-mouse IFN-γ biotin (1 µg/ml) (R46A2) or anti-human IFN-γ (7-B6-1) followed by streptavidin-Alkaline Phosphatase (1 µg/ml) and development with AP conjugate substrate kit (BioRad, UK).

### Intracellular cytokine staining

Murine splenocytes were stimulated for a total of 6 hours with a single pool containing all antigen 85A peptides (2 µg/ml final concentration) or media only (unstimulated control), with the addition of Golgi-Plug (BD) (1 µg/ml) for the final 4 hours. Following surface staining with CD4-APCAlexa780, CD8 PerCPCy5.5, CD62L-PECy7 and CD127 Pacific Blue, cells were fixed with 4% paraformaldehyde and stained intracellularly with TNF-α Alexa488, IL-2 PE and IFN-γ Alexa647 diluted in Perm-Wash buffer (BD).

Frozen macaque PBMCs were thawed and rested overnight in media containing 10 U/ml Benzonase (Sigma). 2×10^6^ PBMCs were restimulated for 6 hours in 96 well U-bottom plates (total volume of 200 µl) with 1 µg/ml anti-human CD49d (9F10), 20 µl anti-human CD28-APC (CD28.2) and 20 µl of anti-human CD107a-PECy5 (eBIOH4A3) in addition to 2 µg/ml antigen 85A peptide pool or DMSO (unstimulated control) with the addition of Golgi-Plug (1 µg/ml) and Golgi-Stop (0.8 µg/ml) for the final 4 hours. Cells were surface stained for CD95-biotin (DX2) followed by strepavidin qDot 565, CD4 eFluro605 (OKT4), Live-Dead Violet (Invitrogen), CD14 eFluro 450 (61D3) and CD20 eFluro 450 (2H7). Cells were fixed with 4% paraformaldehyde and stained intracellularly for TNF-α FITC (MAb11), IL-2 PE (MQ1-17H12), IFN-γ PECy7 (4S.B3), CD3 Alexa700 (SP34-2) and CD8-APC Alexa780 (RPA-T8) diluted in Perm-Wash.

Sample acquisition was performed on a LSR II and data analyzed in FlowJo (TreeStar), Pestle v1.6 and Spice v 4.3 [41] with all final figures made in Canvas X (ACD Systems).

### Statistical methods

All graphs and statistical analysis were created in Prism v5.0c (Graphpad), data were log transformed and a one-way or two-way analysis of variance performed; figure legends denote the test performed for each experiment. Where IMX313 was shown to induce a significant effect, a Kruskal Wallace (one-way ANOVA) or Bonferroni test (two-way ANOVA) was performed and p values stated in the figure legend.

For analysis of macaque peptide pool data, Kruskal-Wallis tests were used to compare the responses from animals at baseline (pre-immunization) with those at all timepoints after immunization. Parametric models estimating the effect of IMX313 modeled log(number of IFN-γ secreting cells, detected by ELISpot) as an outcome variable, because of the approximate log-normal distribution of ELISpot counts in these animals (not shown). Conditions with no spots were scored as having 1 spot, which is the limit of detection of the assay. Modeling of *Log(ELISPOT)∼Effect of IMX313+effect of Timepoint+effect of peptide pool* allowed estimation of an overall effect of IMX313. Assumptions include (i) the responses to the peptide pools are independent, (ii) the effect IMX313 is similar over all peptide pools, (iii) the effect of IMX313 is similar over timepoints, (iv) effect of IMX313 is similar across animals. Analyses used R2.1.1 for Windows and Graph Pad Prism software.

## Supporting Information

Figure S1 **Immune responses to MVA-Ag85A and MVA-Ag85A IMX313 in macaques.** Shown are individual responses to seven peptide pools (A–G) and a 66 peptide pool (‘Pool’) in five animals receiving MVA-Ag85A IMX313 (+) and four animals receiving MVA-Ag85A control (o) before vaccination (shown at t = 0), and at 1,3,5,7,9 and 13 weeks after the first vaccination (at t = 0, dashed vertical line); a second vaccination was given at week 6 (dashed vertical line). Geometric means are shown at each time point, joined by a solid line (Ag85A) or a dashed line (Ag85A IMX313). Conditions with no detectable response are shown as having 1 spot (see Methods). p values test the hypothesis that responses in the Ag85A-IMX313 post vaccination exceed those in the Ag85A group (Krustal-Wallis test).
(TIF)

Figure S2 **IMX313 does not induce activation of APCs.** Balb/c mice were immunized i.d. with 10^6^ PFU MVA-Ag85A (circles) or MVA-Ag85A IMX313 (squares) or unvaccinated (diamonds) and draining auricular lymph nodes harvested 3 days later for analysis by flow cytometry. Organs were diced into small pieces and digested at room temperature for 30 minutes in PBS containing 0.3 mg/ml collagenase-dispase (Roche) and 0.02 mg/ml DNAse. For initial characterization of MVA vaccinated mice, LNs were surface stained for CD80-FITC, CD11c PE, CD8 PerCPCy5.5, B220-PECy7, F4/80 Pacific Blue, CD103-biotin, streptavidin qDot 565, CD4-qDot 655, CD11b Alexa-700, MHC Class II APC-Alexa780 and Live-Dead Aqua prior to fixation and intracellular staining with CD207-APC (eBioscience) in perm-wash buffer. **A**) FACS plots demonstrate the gating strategy to classify the population of CD11c^+^B220^−^CD11b^+^MHC II^int^ DCs relative to plasmacytoid DCs (pDCs), lymph node (LN) resident DCs, or skin migrating DCs isolated from the draining auricular nodes (top panel) or non-draining inguinal and popliteal nodes (bottom panel) three days after id vaccination. CD11b^+^MHC II^int^ DCs were overlayed over the population of LN resident DCs to investigate expression of CD4 and CD8, or overlayed over the population of skin migrating DCs to investigate the expression of CD103 and CD207. Histograms represent the expression of either F4/80 (left) or CD80 (right) on LN resident (grey filled line), skin migrating (black dashed line) or CD11b^+^MHC II^int^ (black bold line) DCs. Graphs represent the percentage of live cells (**B**) or total number (**C**) of B cells (B220^+^CD11c^−^), CD4 (CD4^+^CD8^−^), CD8 (CD8^+^CD4^−^), DCs (CD11c^+^) and CD11c^−^CD11b^+^. CD11c^+^ MHC II^+^ cells were further subdivided into plasmacytoid DCs (pDCs) (B220^+^), CD11b^+^ MHC II^int^, LN resident (CD11b^−^, MHC II^int^) or skin migrating (MHC II^hi^) with graphs representing the percentage (**D**) or total number of each population (**E**). In a separate experiment, auricular LNs from Balb/c mice immunized id with 10^6^ PFU of either MVA-GFP (triangle), MVA-Ag85A (circles), MVA-Ag85A IMX313 (square) or unvaccinated (diamonds) were analyzed at day 3 days by flow cytometry. Digested lymph nodes were surface stained for CD80-FITC, CD11c-PE, CD8-PerCPCy5.5, B220-PeCy7, CD4-qDot650, CD11b-Alexa700, MHC Class II APC with the addition of DAPI prior to acquisition of samples on the LSR II. Graphs represent the percentage (**F**) and total number (**G**) of CD11b^+^MHC^int^ cells or surface expression level of CD80 (**H**) or MHC Class II fluorescence (**I**) on CD11b^+^MHC II^int^ cells. In all graphs, bars represent the mean per group with each individual animal displayed as a single point.
(TIF)

Figure S3 **Expression of activation markers on IFN-g^+^ CD4^+^ or CD8^+^ T cells 3 days after immunization.** Balb/c mice were vaccinated id with 10^6^ PFU MVA85A or MVA85A-IMX313 and the draining auricular lymph node harvested 3 days later. After 6 hours of restimulation with the total 85A peptide pool, cells were surface stained with CD69-FITC, CD44-PE, CD8-PerCPCy5.5, CD62L-PECy7, CD127 Pacific Blue, CD122-bi followed by streptavidin dQot565 (Invitrogen), CD4-eFluro 650 and CD25-Alexa700 prior to fixation and intracellular staining for IFN-γ Alexa647. Cells were subdivided into either CD4^+^ T cells (**A**) or CD8^+^ T cells (**B**) prior to analysis of IFN-γ and surface marker expression. Data was analyzed with a two-way analysis of variance and no statistically significant difference was observed for any marker investigated.
(TIF)

Table S1 **Ag85A peptide pools.** The table lists the separation of the 66 individual 20 mer peptide (overlapping by 10) spanning the length of Ag85A into the 7 peptide pools labelled A to G.
(DOCX)

Table S2 **Amino acid sequences of C4bp oligomerization domains.** Using a Clustal V Method of alignment (MegAlign, DNASTAR Lasergene software), bold letters represents homologous amino acids between chicken, IMX313 and human protein sequences. Grey letters represent the amino acid changes between human and the predicted rhesus macaque sequence (accession no. XP_002801994). Italic letters represent the major sequence change made to chicken version 1 in construction of IMX313, to reduce sequence homology to the human sequence.
(DOCX)
