# Supplementary figures and images for: Fusion of the Mycobacterium tuberculosis Antigen 85A to an Oligomerization Domain Enhances Its Immunogenicity in Both Mice and Non-Human Primates
Source: PLoS One. 2012 Mar 28;7(3):e33555. doi: 10.1371/journal.pone.0033555 (PMC3314664; doi:10.1371/journal.pone.0033555)

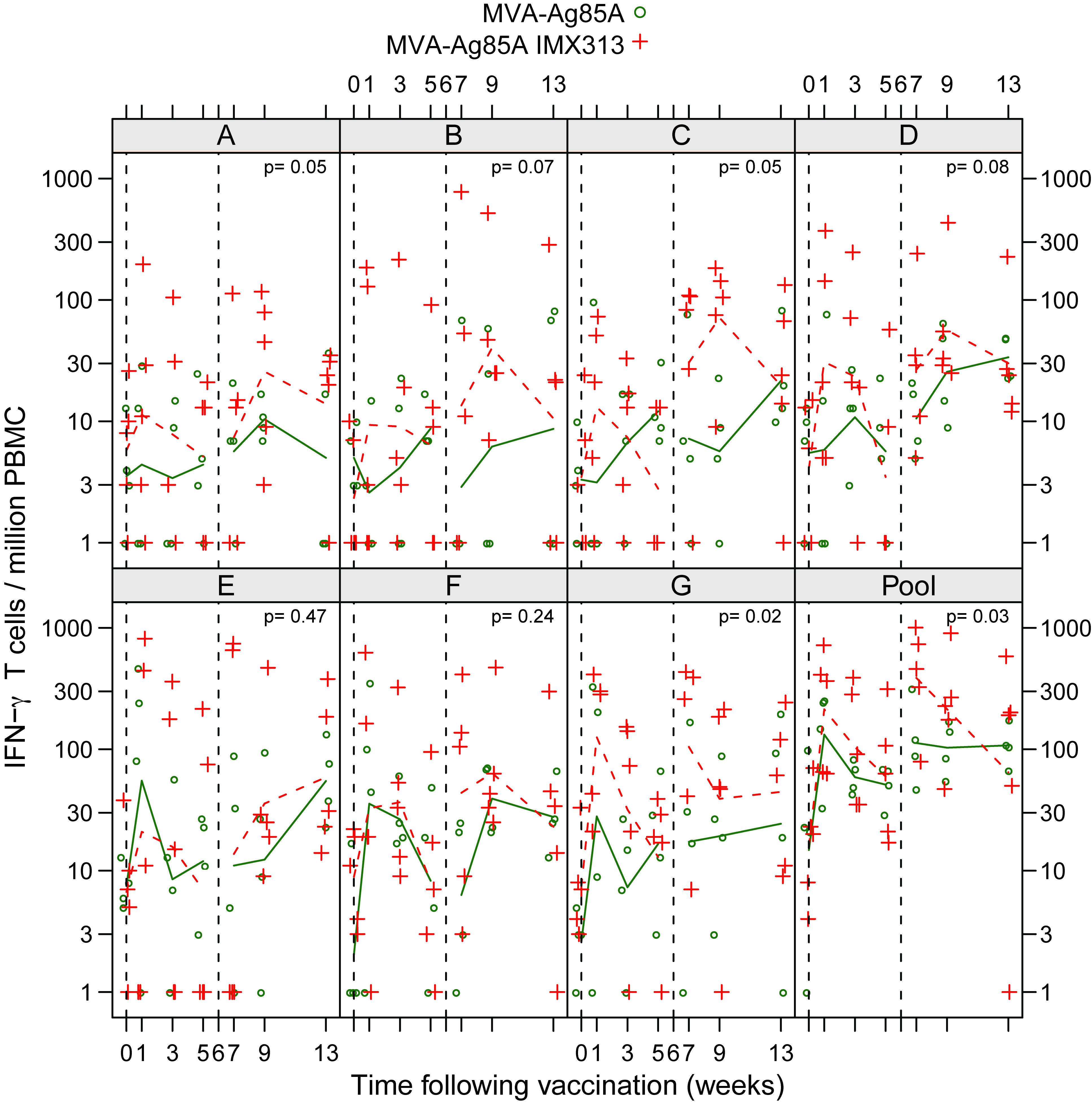

Supplement: Figure S1 — Immune responses to MVA-Ag85A and MVA-Ag85A IMX313 in macaques. Shown are individual responses to seven peptide pools (A–G) and a 66 peptide pool (‘Pool’) in five animals receiving MVA-Ag85A IMX313 (+) and four animals receiving MVA-Ag85A control (o) before vaccination (shown at t = 0), and at 1,3,5,7,9 and 13 weeks after the first vaccination (at t = 0, dashed vertical line); a second vaccination was given at week 6 (dashed vertical line). Geometric means are shown at each time point, joined by a solid line (Ag85A) or a dashed line (Ag85A IMX313). Conditions with no detectable response are shown as having 1 spot (see Methods). p values test the hypothesis that responses in the Ag85A-IMX313 post vaccination exceed those in the Ag85A group (Krustal-Wallis test). (TIF) [file pone.0033555.s001.tif]

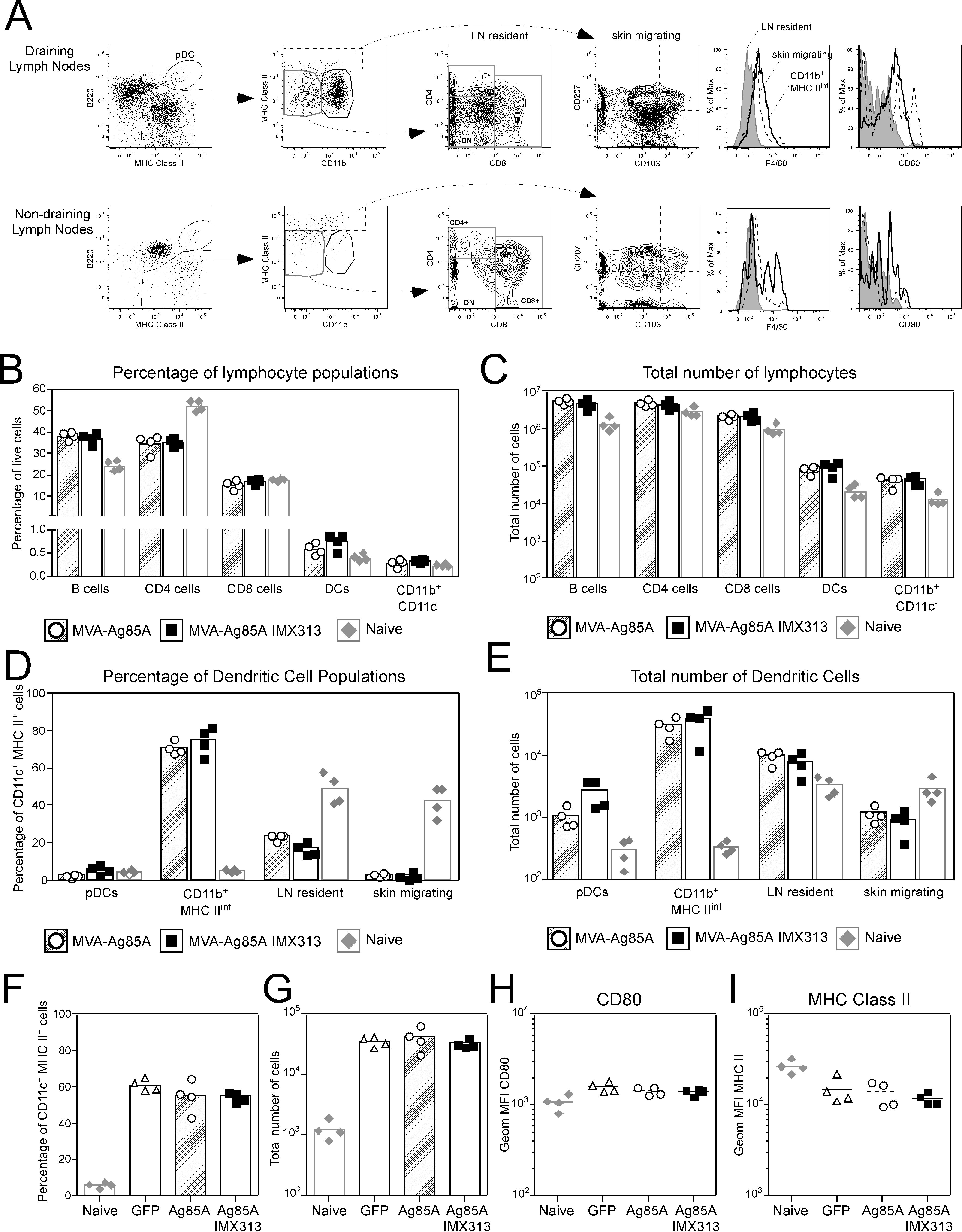

Supplement: Figure S2 — IMX313 does not induce activation of APCs. Balb/c mice were immunized i.d. with 106 PFU MVA-Ag85A (circles) or MVA-Ag85A IMX313 (squares) or unvaccinated (diamonds) and draining auricular lymph nodes harvested 3 days later for analysis by flow cytometry. Organs were diced into small pieces and digested at room temperature for 30 minutes in PBS containing 0.3 mg/ml collagenase-dispase (Roche) and 0.02 mg/ml DNAse. For initial characterization of MVA vaccinated mice, LNs were surface stained for CD80-FITC, CD11c PE, CD8 PerCPCy5.5, B220-PECy7, F4/80 Pacific Blue, CD103-biotin, streptavidin qDot 565, CD4-qDot 655, CD11b Alexa-700, MHC Class II APC-Alexa780 and Live-Dead Aqua prior to fixation and intracellular staining with CD207-APC (eBioscience) in perm-wash buffer. A) FACS plots demonstrate the gating strategy to classify the population of CD11c+B220−CD11b+MHC IIint DCs relative to plasmacytoid DCs (pDCs), lymph node (LN) resident DCs, or skin migrating DCs isolated from the draining auricular nodes (top panel) or non-draining inguinal and popliteal nodes (bottom panel) three days after id vaccination. CD11b+MHC IIint DCs were overlayed over the population of LN resident DCs to investigate expression of CD4 and CD8, or overlayed over the population of skin migrating DCs to investigate the expression of CD103 and CD207. Histograms represent the expression of either F4/80 (left) or CD80 (right) on LN resident (grey filled line), skin migrating (black dashed line) or CD11b+MHC IIint (black bold line) DCs. Graphs represent the percentage of live cells (B) or total number (C) of B cells (B220+CD11c−), CD4 (CD4+CD8−), CD8 (CD8+CD4−), DCs (CD11c+) and CD11c−CD11b+. CD11c+ MHC II+ cells were further subdivided into plasmacytoid DCs (pDCs) (B220+), CD11b+ MHC IIint, LN resident (CD11b−, MHC IIint) or skin migrating (MHC IIhi) with graphs representing the percentage (D) or total number of each population (E). In a separate experiment, auricular LNs from Balb/c mice immunized [file pone.0033555.s002.tif]

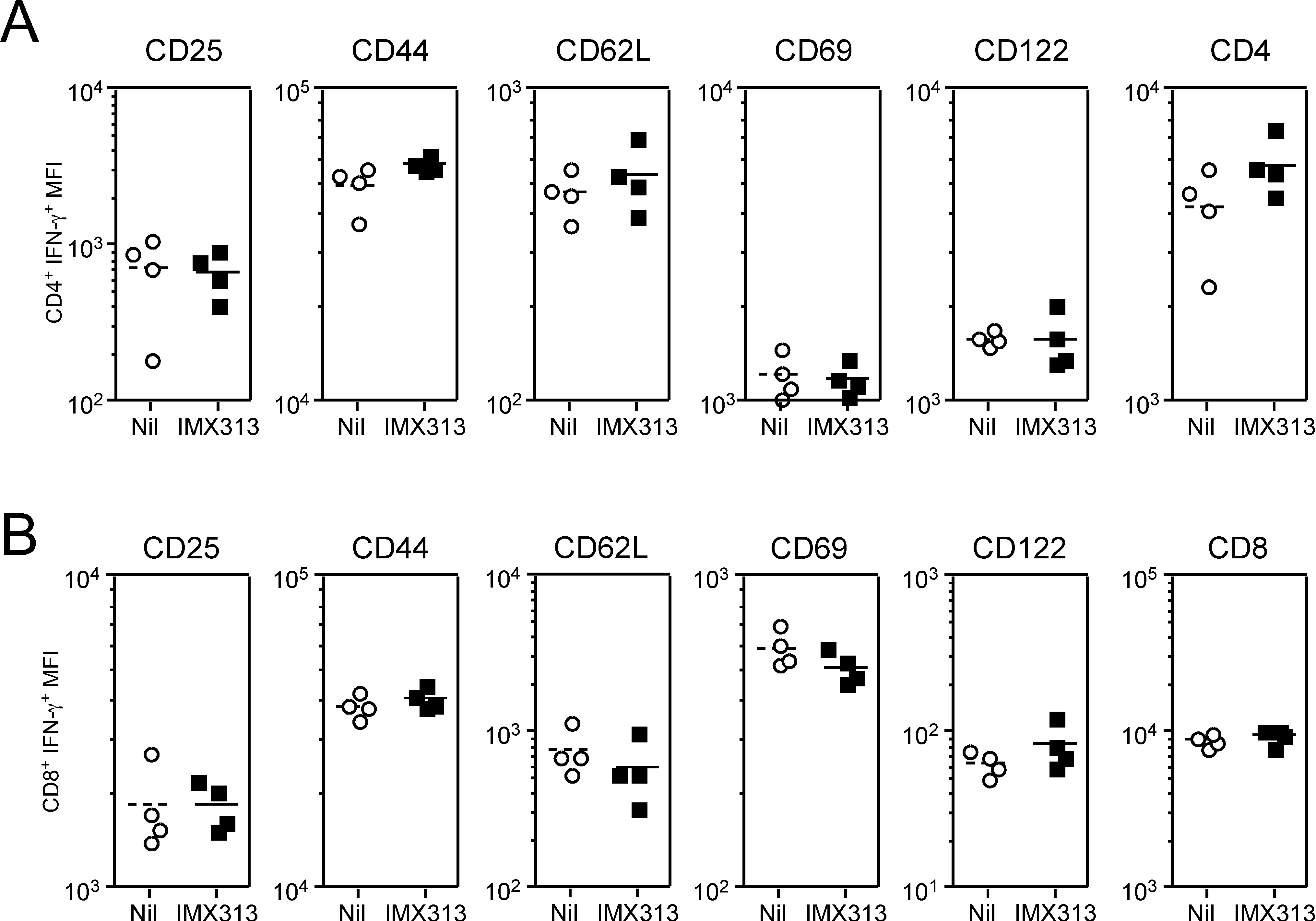

Supplement: Figure S3 — Expression of activation markers on IFN-g+ CD4+ or CD8+ T cells 3 days after immunization. Balb/c mice were vaccinated id with 106 PFU MVA85A or MVA85A-IMX313 and the draining auricular lymph node harvested 3 days later. After 6 hours of restimulation with the total 85A peptide pool, cells were surface stained with CD69-FITC, CD44-PE, CD8-PerCPCy5.5, CD62L-PECy7, CD127 Pacific Blue, CD122-bi followed by streptavidin dQot565 (Invitrogen), CD4-eFluro 650 and CD25-Alexa700 prior to fixation and intracellular staining for IFN-γ Alexa647. Cells were subdivided into either CD4+ T cells (A) or CD8+ T cells (B) prior to analysis of IFN-γ and surface marker expression. Data was analyzed with a two-way analysis of variance and no statistically significant difference was observed for any marker investigated. (TIF) [file pone.0033555.s003.tif]
