# Supplementary material for: Fusion of the Mycobacterium tuberculosis Antigen 85A to an Oligomerization Domain Enhances Its Immunogenicity in Both Mice and Non-Human Primates
Source: PLoS One. 2012 Mar 28;7(3):e33555. doi: 10.1371/journal.pone.0033555 (PMC3314664; doi:10.1371/journal.pone.0033555)
Supplement: Table S1 — Ag85A peptide pools. The table lists the separation of the 66 individual 20 mer peptide (overlapping by 10) spanning the length of Ag85A into the 7 peptide pools labelled A to G. (DOCX) [file pone.0033555.s004.docx]

**Table S1: Ag85A peptide pools**

| **Pool** | **Peptides** |
| --- | --- |
| **A** | p1, p15, p29, p43, p57, p6, p20, p34, p48, p62 |
| **B** | p3, p17, p31, p45, p59, p8, p22, p36, p50, p64 |
| **C** | p5, p19, p33, p47, p61, p10, p24, p38, p52, p66 |
| **D** | p7, p21, p35, p49, p63, p12, p26, p40, p54 |
| **E** | p9, p23, p37, p51, p65, p14, p28, p42, p56 |
| **F** | p11, p25, p39, p53, p2, p16, p30, p44, p58 |
| **G** | p13, p27, p41, p55, p4, p18, p32, p46, p60 |
