# Supplementary material for: Fusion of the Mycobacterium tuberculosis Antigen 85A to an Oligomerization Domain Enhances Its Immunogenicity in Both Mice and Non-Human Primates
Source: PLoS One. 2012 Mar 28;7(3):e33555. doi: 10.1371/journal.pone.0033555 (PMC3314664; doi:10.1371/journal.pone.0033555)
Supplement: Table S2 — Amino acid sequences of C4bp oligomerization domains. Using a Clustal V Method of alignment (MegAlign, DNASTAR Lasergene software), bold letters represents homologous amino acids between chicken, IMX313 and human protein sequences. Grey letters represent the amino acid changes between human and the predicted rhesus macaque sequence (accession no. XP_002801994). Italic letters represent the major sequence change made to chicken version 1 in construction of IMX313, to reduce sequence homology to the human sequence. (DOCX) [file pone.0033555.s005.docx]

**Table S2: Amino acid sequences of C4bp oligomerization domains**

|  | **Sequence** |
| --- | --- |
| **Chicken v1** | KKQGDADV**C**GE**V**AYIQSVVSD**C**HVPT*AELR*TL**LE**IR**KL**F**LEI**QK**L**KVELQ-G----**L**S**KE** |
| **Chicken v2** | KKQGDADV**C**GE**V**AYIQSVVSD**C**HVPT***EDVK***TL**LE**IR**KL**F**LEI**QK**L**KVELQ-G----**L**S**KE** |
| **IMX313** | KKQGDADV**C**GE**V**AYIQSVVSD**C**HVPT*AELR*TL**LE**IR**KL**F**LEI**QK**L**KVELQ-G----**L**S­**KE** |
| **Human** | E---TPEG**C**EQ**V**L­TGKRLM-Q**C**LPNP***EDVK***MA**LE**VY**KL**S**LEI**EQ**L**ELQRDSARQST**L**D**KE**L |
| **Predicted macaque** | E---TPEG**C**EQ**V**LAGKRLM-Q**C**LPNP***EDVK***MA**LE**VY**KL**S**LEI**EQ**L**ELQRDRAR**Q**ST**L**D**KE**L |
